# Birth attendance and magnitude of obstetric complications in Western Kenya: a retrospective case–control study

**DOI:** 10.1186/1471-2393-14-311

**Published:** 2014-09-08

**Authors:** Wilson N Liambila, Shiphrah N Kuria

**Affiliations:** Population Council, P.O. Box 17643, Nairobi, 00500 Kenya; The Division of Reproductive Health, Department of Family Health, Ministry of Health, Mbagathi Road (Old), P.O. Box 43319, Nairobi, Kenya

**Keywords:** Birth attendance, Child birth related complications, Risk factors, Postpartum women, Community level, Skilled attendants, Unskilled attendants, Kenya

## Abstract

**Background:**

Skilled birth attendance is critical in the provision of child birth related services. Yet, literature is scanty on the outcomes of child birth related complications in situations where majority of women deliver under the care of non-skilled birth attendants compared to those who are assisted by skilled providers. The study sought to assess the nature of childbirth related complications among the skilled and the non-skilled birth attendants in Western Kenya.

**Methods:**

A case–control study was conducted among women aged 15–49 years at the household. Controls were individually matched to cases on the basis of age and socio-economic status. A total of 294 cases and 291 controls were interviewed. Data were collected on various demographic and socio-economic characteristics and women’s perception on the quality of care. All independent variables were analysed initially in bivariate models and those that were significantly associated with obstetric complications were included in multiple logistic regression model in order to control for confounding factors. Odds ratios (ORs), with 95% confidence intervals, were computed to show the association between the occurrence, magnitude and the extent to which child birth related complications were managed.

**Results:**

Demographic and socio-economic characteristics of the cases and controls were similar. About 52% of the deliveries were assisted by skilled birth attendants while non-skilled providers attended to 48% of them. The odds of the occurrence of obstetric complications were greater among the women who were attended to by skilled providers in health facilities: adjusted odds ratio (AOR): 1.32 (CI 0.95, 1.84) than among those who were assisted by unskilled birth attendants, AOR 0.76 (CI 0.55, 1.06). Undignified care, high delivery and transport costs and fear of hospital procedures such as HIV tests and mishandling of the placenta were cited as some of the barriers to facility deliveries.

**Conclusion:**

Skilled birth attendants in facilities were associated with higher odds of the occurrence of obstetric complications compared to deliveries that were assisted by non-skilled attendants at home. Women cited many barriers which need to be addressed in order to improve their access to skilled providers for delivery and in managing obstetric complications.

## Background

Kenya still experiences high maternal and perinatal morbidity and mortality. For instance, in 2008/09 maternal mortality ratio was estimated to be 488/100,000 live births [1] against a millennium development target of 147/100,000 live births by 2015. The percentage of births assisted by a skilled provider is still low in Kenya. Just under 44% of births in Kenya are delivered under the supervision of a skilled birth attendant (SBA) who could be a nurse or midwife, doctor or clinical officer. Despite concerted efforts by the Government to increase the proportion of deliveries conducted by SBAs, non-skilled birth attendants (non-SBAs) namely traditional birth attendants (TBAs), neighbours, friends or self/no one present deliveries continue to play an important role in child birth activities. For instance, about 28% of all deliveries in Kenya are conducted by TBAs. This accounts for about 50% of deliveries performed by non-SBAs. Relatives and friends assist with 21% of births, and for seven percent of births, mothers do not receive any form of assistance [2].

There is a correlation between regions with a higher proportion of skilled attendance during delivery and low maternal mortality ratios as well as perinatal mortality rates [3]. For instance, according to the World Health Organization (WHO) Global Strategy for Women’s and Children’s Health, the 2010 maternal mortality as well as neonatal mortality rates in countries where women deliver at home under skilled attendance are comparable to those in countries where women mainly deliver in health facilities [4]. Little is known about the association of health outcomes of women who deliver using non-SBAs compared to those who deliver under the care of SBAs [5]. Literature is also scanty on the outcomes of child birth related complications in situations where TBAs conduct a substantial proportion of deliveries [6].

The main aim of the study was to assess the nature of childbirth related complications between the skilled birth attendants (SBAs) and the non-skilled birth attendants (non-SBAs). A secondary aim of the study was to assess the role of socio economic, demographic and health related factors in the occurrence and management of childbirth related complications among women in Western Kenya who had delivered in health facilities and at home.

The study focused on Western Kenya, which has low facility based deliveries (25.8%) compared to the national average of 43.8% as reported in *the Kenya’s Demographic and Health Survey 2008–09,* referred to earlier.

## Methods

### Study design

The study used a case–control design. It was conducted among women aged 15-49 years at the community level in the Western region of Kenya for a period of four months between August 2013 and November 2013.

### Study area

The study was implemented in Bungoma County and Lugari Sub-County of Western Kenya. According to the 2009 Kenya Population and Housing Census, Bungoma County with a land mass of 2069 sq.km has an estimated population of 1,630,934 [7]. In terms of health services, it has 12 hospitals providing Comprehensive Emergency Obstetric care (CEmOC). There are 14 health centres, 106 dispensaries, and 4 nursing homes. The situation regarding the coverage of health facilities in Lugari Sub-county (situated in Kakamega County) is a little different from that of Bungoma County. Lugari Sub-county, with an estimated population of about 300,000 has only one facility (Lumakanda District Hospital) providing CEmOC and women requiring these services are often referred to health facilities which are located outside the sub-county. Besides Lumakanda District Hospital, Lugari Sub-County has two other hospitals which are not well equipped (for instance, they do not provide blood transfusion and caesarean section services). The sub-county has eight health centres, 29 dispensaries and three nursing homes. None of these are able to perform all the Basic Emergency Obstetric Care (BEmOC) functions [8].

Bungoma County and Lugari Sub-County were selected due to a number of reasons including the fact that the proportion of home deliveries and the percentage of births delivered by unskilled providers (estimated at 74.2%) are high [9]. Other reasons for choosing the study sites included: presence of an active network of TBAs, having an active network of community midwives and both sites share a similar agro-ecological zone and demographic and socio-economic characteristics [10].

### Target population

A case was defined as any woman aged 15–49 years who delivered within the past 12 months preceding data collection who suffered from a complication while in labour, during delivery or within 42 days after delivery that either necessitated treatment, referral or hospitalization. The period of 12 months was used in order to capture information regarding the complications that could have persisted after delivery and postpartum period and to provide a good window for the study to achieve the desired sample sizes and still minimize the possibility of recall bias. Key complications that were assessed can be grouped into direct and indirect causes of maternal mortality [11]. The direct causes include fever/lower abdominal pain (puerperal sepsis or infection), haemorrhage, headache/blurred vision, swollen face, hands, legs, tiredness, convulsions (fits) or eclampsia/preeclampsia, retained placenta, ruptured uterus and obstructed or prolonged labour. The indirect causes of maternal mortality include malaria, HIV/AIDS, tuberculosis (TB), severe anaemia and heart conditions. For women who developed complications after delivery, the study sought information on whether the complications were successfully treated, worsened or persisted. Controls were defined as women aged 15–49 years who delivered within the past 12 months preceding data collection but didn’t have any complications.

Besides the causes of maternal mortality referred to above, women also experience severe maternal morbidities that may sometimes complicate into severe disability or death. Examples of maternal morbidity are: anaemia (referred to earlier), maternal depression, infertility, obstetric fistula, uterine rupture and scarring and genital and uterine prolapse [12].

### Sample size for the case–control study

From previous studies reported by World Health Organization and the British Medical Bulletin [13], about 15% of women experience pregnancy and childbirth related complications. In order to calculate the sample size required for an individually matched case control study, the formula applied was based on the null hypothesis which, in the context of this study, stated that the population odds ratio is equal to one and that there is no difference in the exposure rates of controls and cases to the risk factors being investigated. A total of 300 subjects were targeted in each arm. The target sample took into account 15% non-response rate, which could be due to refusal or unavailability [14]. Out of 600 women (300 cases and 300 controls) that were targeted to take part in the study, a total of 294 cases (98% response rate) and 291 controls (97% response rate) participated in the study. The numbers of locations from where study participants were identified were chosen in such a way that the selection reflected the population ratio of the two study sites (Bungoma and Lugari) based on the 2009 census. Thus, as shown in Table 1, eight locations namely, Webuye, Sitikho, Miendo, Kibabii, Kimaeti, Kibuke, Kabuchai and North Nalondo were selected from Bungoma County and two locations (Lumakanda and Kongoni) from Lugari Sub-County. The distribution of cases and controls within the selected locations were therefore similar.

**Table 1:** **Names of locations surveyed in the study sites by number of cases and controls**

Variable	Cases (N = 294)		Controls (N = 289)		P-value
**Survey sites**	**N**	**%**	**n**	**%**	1.000
Lumakanda*	26	8.8	26	8.9	
Kongoni*	14	4.7	14	4.8	
Webuye**	45	15.3	45	15.5	
Sitikho**	15	5.1	12	4.1	
Miendo**	27	9.2	26	8.9	
Kibabii**	19	6.4	19	6.5	
Kimaeti**	30	10.2	29	10	
Kibuke**	52	17.6	52	17.9	
Kabuchai**	17	5.8	17	5.8	
North Nalondo**	50	16.9	51	17.5	
County total					
Lugari Sub-County (Kakamega County)	41	50.6	40	49.4	0.957
Bungoma County	254	50.0	251	50.0	0.957

*Locations selected in Lugari sub-County (Kakamega County) **Locations selected in (Bungoma County).

### Selection and matching of cases and controls

The study was conducted at the community level through individual matching of controls to cases on the basis of age and the geographical location of the villages and households where the study subjects were recruited. Matching was necessary in order to increase precision of estimates and reduce the standard error and to achieve narrower confidence intervals [15]. Controls were individually matched to the cases on the basis of age and if both shared comparable socio-economic status such as type of housing and level of education.

### Sampling procedures and data collection

Data were collected at the household level through structured interviews administered by trained research assistants. The survey tool was translated from English into Kiswahili and pre-tested for consistency before data collection. In each of the selected locations, the first house households were selected through a simple random process. Once a household had been selected, then the research assistants used a screening tool to identify an eligible woman who had experienced an obstetric complication in the past 12 months (a case). For each case identified and recruited, selection and recruitment of an appropriate control from the neighbourhood within the same location was conducted concurrently. The selected controls had similar demographic and socio-economic characteristics to the recruited cases. Thus, the procedure for identifying and recruiting cases and controls was repeated in each location until the required sample size was achieved. Once an eligible woman was identified in the selected location, written informed consent to participate in the study was obtained and she was then recruited as a case. Information was collected on demographic and socio-economic characteristics such as age, number of pregnancies and miscarriages, marital status, highest level of schooling, religion, main occupation, type of housing as well as the nature of complications women experienced, main reason for home deliveries, condition of baby after birth and information on women’s perception on the quality of care received from their attendants or providers. Information was also obtained on the perception of women regarding the occurrence of disrespect and abuse of women while seeking various reproductive health services both at home and in health facilities as well as on details surrounding childbirth in terms of the type of attendants who conducted the deliveries and the place of delivery.

### Data analysis

Data was entered using Epi Data Software version 3.1, cleaned and then exported to STATA statistical software version 11 for analysis. Categorical variables were expressed as frequency and percentage and were tested for significance using a chi-square test. Cross tabulations of independent variables with the occurrence or experiences of obstetric complications were performed. The independent variables included the use of skilled attendants (community midwives, health facility providers) and unskilled attendants (TBAs, relatives, neighbours, friends or self). All independent variables were analysed initially in bivariate models and those that were significantly associated with obstetric complications (dependent variable) were included in multiple logistic regression models in order to identify confounding factors and to measure the independent effects of each exposure variable on occurrence of complications. The strength of association of selected risk factors for obstetric complications was determined by estimating the odds ratios (ORs) and their 95% confidence intervals (CIs). A probability of less than 0.05 was considered statistically significant. The study was approved by the Kenyatta National Hospital Ethical Review Committee.

The research upon which this paper is based adhered to the STROBE (Strengthening the Reporting of Observational studies in Epidemiology) guidelines as outlined here: http://www.strobe-statement.org.

## Results

### Demographic and socio-economic characteristics of participants

Overall, the demographic and socio-economic characteristics of the cases and controls were similar. The median age for both cases and controls was 26 years (range15-48 years). In both cases and controls, most of the participants were married, protestant/other Christians and were involved in farming/agricultural activities (Table 2). Majority of the cases and controls were in the 25–29 years age group and had experienced at least 4 pregnancies or miscarriages. In both cases and controls, the majority of participants had achieved primary level schooling (both complete and incomplete) with controls having a slightly higher proportion than cases among those who had achieved primary level education (54% of controls versus 45% of cases). Majority of respondents were of low socio-economic status with tinned roof houses and mud walled houses (78% cases and 83% controls).

**Table 2:** **Demographic and socio-economic characteristics of cases and controls**

Variable	Cases (N = 294)		Controls (N = 289)		p-value
Age	n	%	n	%	
< 18 yrs	12	4.1	18	6.3	0.241
19-24 yrs	100	33.9	95	33.0	0.770
25-29 yrs	115	39.0	107	37.2	0.603
30 yrs and over	67	22.7	69	24.0	0.659
**Number of pregnancies and miscarriages**					
1	62	21.1	64	22.2	0.697
2	75	25.4	75	26.0	0.922
3	50	16.9	42	14.6	0.402
4	82	27.8	82	28.5	0.946
>4	25	8.5	27	9.4	0.948
Median No. of pregnancies (Range)	3.0	(1,14)	3.0	(1,12)	
Mean No. of pregnancies (95% CI)	3.3	(3.1, 3.6)	3.2	(3.0, 3.4)	0.443
**Marital status**					
Unmarried/single	28	9.5	28	9.7	0.957
Married	259	87.8	248	86.1	0.357
Divorced/separated/widowed	7	2.4	14	4.8	0.280
**Highest level of schooling**					
No education	38	12.9	38	13.2	0.962
Primary incomplete	76	25.8	84	29.2	0.468
Primary complete	57	19.3	71	24.7	0.142
Secondary incomplete	53	18.0	49	17.0	0.705
Secondary complete	41	13.9	35	12.2	0.49
Tertiary (complete)	27	9.2	13	4.5	0.024
**Religion**					
Catholic	79	26.8	77	26.7	0.951
Protestant/other Christian	195	66.1	202	70.1	0.351
Muslim	11	3.7	4	1.4	0.072
No religion	7	2.4	4	1.4	0.376
**Main occupation**					
Self employed	67	22.7	42	14.6	0.011
Farming/agriculture	138	46.8	146	50.7	0.385
Skilled labour	21	7.1	28	9.7	0.268
Unskilled labour	25	8.5	33	11.5	0.239
Professionals e.g. teacher, engineer etc.	27	9.2	17	5.9	0.131
Not employed/housemaid/student	14	4.7	21	7.3	0.203
**Type of wall material**					
Mud/Earth	228	77.8	240	83.0	0.112
Bricks	57	19.5	41	14.2	0.09
Blocks	8	2.7	6	2.1	0.607
Other (stones,wood,iron)	0	0	2	0.4	0.314

### Occurrence of obstetric complications and demographic and socio-economic factors

Overall, demographic and socio-economic factors assessed (Table 3) seemed not to be significantly associated with the risk of the occurrence of obstetric complications.

**Table 3:** **Risk of obstetric complications by demographic and pregnancy-related factors**

Variable	Cases (N = 294)		Controls (N = 289)		OR (95% CI)*	p-value
Age	N	%	N	%		
< 18 yrs	12	4.1	18	6.3	0.59 (0.25,1.40)	0.228
19-24 yrs	100	33.9	95	33.0	1.58 (0.72,3.45)	0.253
25-29 yrs	115	39.0	107	37.2	1.61 (0.74,3.50)	0.228
30 yrs and over	67	22.7	69	24.0	1.45 (0.65,3.26)	0.359
**Number of pregnancies and miscarriages**						
1	62	21.1	64	22.2	0.97 (0.64,1.48)	0.887
2	75	25.4	75	26.0	1.03 (0.64,1.66)	0.895
3	50	16.9	42	14.6	1.23 (0.72,2.11)	0.453
4	82	27.8	82	28.5	1.03 (0.59,1.79)	0.910
>4	25	8.5	27	9.4	1.00 (0.62,1.63)	0.995
**Marital status**						
Unmarried/single	28	9.5	28	9.7	0.96 (0.55,1.66)	0.878
Married	259	87.8	248	86.1	0.76 (0.46, 1.24)	0.266
Divorced/separated/widowed	7	2.4	14	4.8	0.48 (0.19,1.21)	0.118
**Highest level of schooling**						
No education	38	12.9	38	13.2	1.09 (0.63,1.88)	0.755
Primary incomplete	76	25.8	84	29.2	0.76 (0.46, 1.24)	0.266
Primary complete	57	19.3	71	24.7	0.88 (0.55,1.40)	0.577
Secondary incomplete	53	18.0	49	17.0	1.18 (0.72,1.94)	0.514
Secondary complete	41	13.9	35	12.2	1.28 (0.74,2.21)	0.379
Tertiary (complete)	27	9.2	13	4.5	2.27 (1.09,4.70)	0.028
**Religion**						
Catholic	79	26.8	77	26.7	1.06 (0.73,1.54)	0.747
Protestant/other Christian	195	66.1	202	70.1	0.63 (0.44, 0.91)	0.014
Muslim	11	3.7	4	1.4	2.85 (0.89,9.10)	0.077
No religion	7	2.4	4	1.4	1.81 (0.52,6.29)	0.349
**Main occupation**						
Self employed	67	22.7	42	14.6	1.69 (1.08, 2.65)	0.023
Farming/agriculture	138	46.8	146	50.7	0.71 (0.51, 1.02)	0.060
Skilled labour	21	7.1	28	9.7	0.79 (0.43,1.46)	0.459
Unskilled labour	25	8.5	33	11.5	0.80 (0.45,1.42)	0.446
Professionals e.g. teacher	27	9.2	17	5.9	1.68 (0.88,3.32)	0.118
Other	14	4.7	21	7.3	0.71 (0.34,1.44)	0.339
**Type of wall material**						
Mud/Earth	228	77.8	240	83.0	0.57 (0.37,0.87)	0.08
Bricks	57	19.5	41	14.2	1.81 (1.16,2.83)	0.08
Blocks/wood/stones	8	2.7	8	2.7	1.51 (0.52,4.44)	0.445

*This is crude odds ratio (non-adjusted).

### Skilled and non-skilled birth attendants and place of delivery

Skilled birth attendants (SBAs) in health facilities conducted most of the deliveries (40.8% of cases and 32.6% of controls). These were followed by non-skilled birth attendants (non-SBAs) especially the traditional birth attendants who conducted 34.3% of deliveries for cases and 29.9% among controls (Table 4). Women in the control group were more likely to deliver on their own compared to women who were cases (p < 0.05). Cases were more likely to deliver in health facilities than at home while controls were more likely to deliver at home (p < 0.05).

**Table 4:** **Distribution of deliveries by skilled and non-skilled birth attendants and place of delivery**

Delivery conducted by:	Case (N = 294)	(%)	Control (N = 288)	(%)	p-value
***Skilled births attendants (SBAs)***					
Community midwife	34	15.7	49	17.0	0.655
Health provider at health facility	120	40.8	94	32.6	0.041
***Non-skilled births attendants (non-SBAs)***					
Traditional birth Attendant (TBA)	101	34.4	86	29.9	0.246
Relative/neighbour/friend	7	2.4	13	4.5	0.158
Self/own	20	6.8	46	16.0	<0.001
**Place of delivery**	**Case (N = 282)**	**(%)**	**Control (N = 288)**	**(%)**	
Facility	120	42.6	94	32.6	0.038
Home	162	57.4	194	67.4	0.038
Type of attendant during delivery	Case (N = 294)	%	Control (N = 291)	%	
Non-skilled	129	43.7	148	50.9	0.542
Skilled	166	56.3	143	49.1	0.084

Non-skilled providers were more likely to conduct deliveries among the control group (51%; n = 148) compared to the cases (44%; n = 129). Skilled providers were more likely to conduct deliveries among the cases (56%; n = 166) compared to the control group (49.1%; n = 143). Overall, about 52% (n = 312) of all deliveries surveyed were assisted by skilled providers while unskilled attendants (traditional birth attendants, neighbours, relatives and friends including self) attended to 48% of the deliveries.

### Experiences of women regarding child-birth related complications

**Figure 1:**
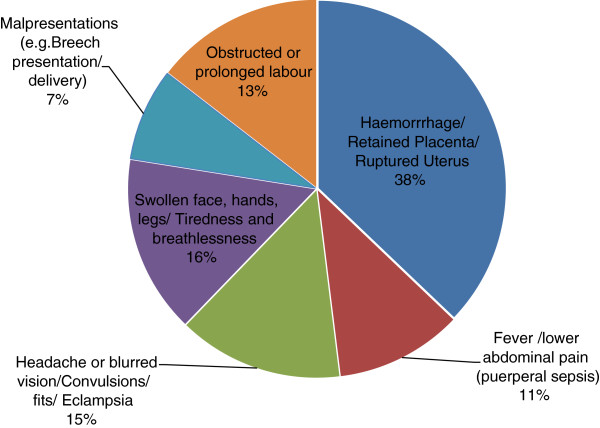
**Proportions of obstetric complications as stated by respondents (N = 295).**

Cases were asked to state the nature of child-birth related complications they had experienced. The main obstetric complications which were mentioned by women who had experienced them are summarised in Figure 1. Haemorrhage was the leading cause of common obstetric complications documented, accounting for 38% of the total complications documented in the study.

### Management and referral of obstetric complications

Study participants who had experienced complications were asked whether their respective providers managed the complications or referred them elsewhere for further management. The management and referral of obstetric complications varied significantly across the levels of care. For instance, 207 out of 289 (72%) cases reported that their complications were successfully managed while 82 (28%) were referred elsewhere for further management. Of the 207 cases whose complications were successfully managed, 45% were managed at home while 55% were managed in health facilities (Table 5). With regard to the management of cases at home or in health facilities, the approach followed was similar. Either the provider or care-giver discussed the problem with the client or relatives and re-assured them or gave drugs to stabilise the patient. Other approaches included counselling the patient or calling for help (i.e. other persons, neighbours, providers, vehicle, ambulance; etc.). With regard to the administration of drugs or performing certain technical procedures such as manual removal of the placenta or retained membranes at home (in case of postpartum haemorrhage), these activities were mainly performed by community midwives.

**Table 5:** **Management and referral of obstetric complications of cases**

Place of delivery	Managed		Referred		P-value
	207	(%)	82	(%)	
Home	93	(44.9)	75	(91.5)	<0.001
Facility	114	(55.1)	7	(8.5)	
**If managed, what did the provider do?**			**N = (190)**		**(%)**
Discussed the problem and reassured patient			36		19.0
Gave drugs/stabilised patient			82		43.1
Advised/counselled patient and gave a follow-up appointment for a check up			27		14.2
Called for help to manage on site			6		3.2
Other			39		20.5
**Total**			**190**		**100.0**

Women who had experienced complications were also asked, *“How soon before, after or during delivery did the complications develop?”* Close to 55% said that the complications had developed in 0–4 hours while 13% said that the complications had developed in 5–8 hours.

### Proportion of women who reported disrespect and abuse

Both cases and controls were asked whether they had experienced any form of disrespect and abuse while in labour, during the process of delivery or immediately after delivery (Table 6). Almost all forms of disrespect and abuse were associated with a greater risk of the occurrence of obstetric complications. In addition, more cases experienced non- dignified care compared to controls (p < 0.05).

**Table 6:** **Proportion of women who reported disrespect and abuse**

Type of abuse* (see questions asked at the bottom of this table)	Case		Control		Odds ratio	P-value*
	293	%	287	%		
Physical abuse	52	(17.7)	37	(12.9)	1.46 (0.92,2.30)	0.105
Non consented care	77	(26.3)	65	(22.6)	1.23 (0.84,1.80)	0.287
Non confidential care	23	(7.8)	13	(4.5)	1.80 (0.89,3.63)	0.095
Non dignified care	32	(10.9)	17	(5.9)	1.96 (0.92,2.30)	0.030
Discrimination	27	(9.2)	28	(9.8)	0.94 (0.54,1.64)	0.824
Abandonment of care	32	(10.9)	25	(8.7)	1.29 (0.74,2.23)	0.371
Detention in facilities	31	(10.6)	21	(7.3)	1.49 (0.84,2.67)	0.173

*a) Physical abuse: Did anyone hit, pinch, slap, or otherwise hurt you as punishment?

*b) Non-consented care*: Were you and/or your partner asked for permission before conducting some procedures while in labour?

*c) Non-confidential care*: Could people other than attendants hear information about you that you would have preferred to be private?

*d) Non-dignified care*: Was anyone rude or verbally abusive to you before/during/after delivery?

*e) Discrimination*: Did anyone comment on your age, wealth, marital status or ethnicity in a way that seemed judgmental?

*f) Abandonment of care*: After being admitted, how long did you have to wait before someone checked on your labour progress?

*g) Detention in facilities or care giver’s home*: Did you have to stay at a care giver’s place longer than necessary due to payment problems?

### Main reasons for home deliveries

Both cases and controls (including those who gave birth in health facilities) were asked to state the most important reason from their perspective as to why women in their local community prefer to deliver at home. All the cases and controls were asked the same question irrespective of the place they delivered. Data were analysed by cases and controls and the type of attendant who assisted during childbirth (Table 7). In addition, further analysis of the data by respondents’ place of delivery revealed significant differences between those who delivered at home and in health facilities. For instance, more women who delivered at home cited preference for TBAs compared to those who delivered in health facilities (p < 0.05). TBAs were preferred because they were viewed as being friendly and caring and provided complementary services such as food, warm beverages, and bathing water to women who have given birth when compared to health facility deliveries.

**Table 7:** **Main reason for home deliveries by cases and controls and type of attendant**

Main reason for home deliveries by cases and controls							
Responses	Case		Control		Total		P-value
	(n = 294)	(%)	(n = 285)	(%)	(n = 579)	(%)	
Is good sign that the woman is strong	30	10.2	28	9.8	58	10.0	0.879
Fear of HIV test, C/S, mishandling of placenta	32	10.9	34	11.9	66	11.4	0.692
Preference for TBAs*	11	3.7	13	4.6	24	4.1	0.621
High transport costs	57	19.4	65	22.8	122	21.1	0.313
Rude or uncooperative H/facility staff	63	21.4	59	20.7	122	21.1	0.83
Poor quality care in health facilities	14	4.8	24	8.4	38	6.6	0.075
High delivery cost in health facilities	16	5.4	2	0.7	18	3.1	0.001*
Poverty & ignorance	53	18.0	58	20.4	111	19.2	0.478
Don’t know	18	6.1	2	0.7	20	3.5	<0.001
**Main reason for home deliveries by type of attendant who assisted the respondent at childbirth**							
**Responses**	**Non-skilled attendant**		**Skilled attendant**		**Total**		**P-value**
	**(n = 272)**	**(%)**	**(n = 307)**	**(%)**	**(n = 579)**	**(%)**	
Is good sign that the woman is strong	26	9.6	32	10.4	58	10.0	0.729
Fear of HIV test, C/S, mishandling of placenta	27	9.9	39	12.7	66	11.4	0.294
Preference for TBAs*	20	7.4	4	1.3	24	4.1	<0.001
High transport costs	47	17.3	75	24.4	122	21.1	0.035
Rude or uncooperative H/facility staff	58	21.3	64	20.8	122	21.1	0.888
Poor quality care in health facilities	13	4.8	25	8.1	38	6.6	0.103
High delivery cost in health facilities	7	2.6	11	3.6	18	3.1	0.485
Poverty & ignorance	62	22.8	49	16.0	111	19.2	0.037
Don’t know	12	4.4	8	2.6	20	3.5	0.235

*TBAs were seen as being friendly and caring and provided complementary services such as food, warm beverages, and warm bathing water to women who had given birth. Respondents also mentioned that TBAs displayed great compassion towards women who were having labour pains by soothing or rubbing their backs.

In addition, more women cited poor quality of care in health facilities as the reason for delivering at home (p < 0.05). Poor quality of care in health facilities encompasses issues such as limited flexibility in choosing freely the best position to deliver (some health providers insist that a woman in labour should lie or deliver a baby while in a particular position), mothers being left to be taken care of by trainee nurses or students and lack of follow up by health facility staff unlike the TBAs who are often available to their clients. Other factors that were cited as contributing to poor quality of care include the fact that women in labour are expected to buy supplies e.g. cotton wool, gloves etc. and aren’t free to choose trusted persons to assist them during childbirth (one is assisted by any staff on duty). In addition, women mentioned that after delivery in the health facilities (particularly the public sector), one isn’t provided with critical supplies and services such food, warm beverages, and bathing water.

A higher proportion of those who were delivered by skilled attendants cited high transport costs (24.4%) compared with those who were delivered by non-skilled attendants (17.3%), (p < 0.05). The other reasons included the fact that delivering at home gives a woman more dignity and is a sign that she is strong, fear of HIV tests, undergoing unnecessary caesarean-section and mishandling of the placenta.

### Quality of care received during antenatal care

Quality of antenatal care was assessed on the basis of the standard practices and expected content of the planned visits. Examples of services assessed were: discussions about birth planning during the ANC visits, administration of iron pills/folate, use of anti-malarial pills, HIV testing and counselling, expected date of delivery (EDD) and tetanus toxoid inoculation among others. We also assessed whether during the antenatal visits, the provider checked the clients’ blood pressure, performed abdominal examination, blood level and listened to the baby’s heartbeat. A review of some of the findings indicates that the proportion of women who developed birth plans was as low as 49.8% for the cases and 48.8% for the controls (Table 8). Women who delivered on their own recorded the lowest proportion (34%) of those who had prepared their birth plans. As expected, more cases (42%) than controls (19%) were referred to health facilities before or after delivery p < 0.001. Overall, only 24% of the cases and 25% of the controls sought targeted antenatal care services at least four times.

**Table 8:** **Quality of care received during antenatal care for both cases and controls**

How many times did you seek ANC this last pregnancy	Cases		Controls		p-value
	N = 281	(%)	N = 272	(%)	
None	1	0.4	4	1.5	0.236
Once	13	4.6	12	4.4	0.903
Twice	25	8.9	37	13.6	0.08
Three times	68	24.2	63	23.2	0.774
Four times	67	23.8	67	24.6	0.829
Five and above	107	38.1	89	32.8	0.186
**Did the provider discuss your birth plan during the ANC visits?**	**N = 291**	**(%)**	**N = 287**	**(%)**	
Yes	157	54.1	157	55.1	0.578
If yes, Did you prepare individual birth plan?	145	49.8	140	48.8	0.795
During your previous ANC visits, did the Provider give you iron pills?	232	79.7	216	75.3	0.199
During the ANC visits, did the Provider give you anti-malarial pills?	258	88.7	252	87.8	0.837
During ANC, did the provider ask if you had received tetanus toxoid?	244	83.8	235	81.9	0.516
Did the provider inform you of your Expected date of delivery?	248	85.2	242	84.3	0.557
While preparing your birth plan, did you involve other persons?	214	73.5	201	70.0	0.457
**IF YES, who IN PARTICULAR did you involve in making/preparing your individual birth plan?**	**N = 214**	**(%)**	**N = 201**	**(%)**	
Father of baby	130	60.7	113	56.2	0.313
Sister	10	4.7	7	3.5	0.520
Mother-in-law	49	22.9	40	19.9	0.486
Mother	16	7.5	31	15.4	0.001
Other (Auntie, friend, etc.)	10	4.7	11	5.4	0.707
**During the antenatal visits did the provider…?**	**N = 291**	**(%)**	**N = 286**	**(%)**	
Check your blood pressure	243	83.5	247	86.4	0.337
Perform abdominal examination	281	96.6	275	96.2	0.793
Discuss blood levels (anaemia)	260	89.3	251	87.8	0.534
Listen to the baby's heartbeat	277	95.2	274	95.8	0.722
Discuss the need for an HIV test/HIV status	261	89.7	247	86.4	0.218
Were you referred to a health facility before or after delivery?	123	42.3	54	18.9	0.000
Have you experienced abnormal leakage of urine since your last delivery?	57	19.6	59	20.6	0.598
ANC: Antenatal care					

### Factors associated with the baby’s condition after birth

As shown in Table 9, babies who were delivered by women in the control group (93%), were significantly more likely to cry immediately after birth (p < 0.01) compared to babies who were delivered by women who were cases (71%). Babies who were delivered by women who were cases were, on the other hand, significantly more likely to be born dead or die immediately they are born (9%) compared to babies who were delivered by women in the control group (0.5%); p < 0.01. Slightly more mothers who were cases and their babies were checked within 48 hours after delivery compared to the mothers and their babies who were in the control group.

**Table 9:** **Condition of baby after birth among cases and controls**

	Case		Control		P-value
Condition of baby after birth	n (227)	(%)	N (183)	(%)	
Cried immediately	162	(71.4)	171	(93.4)	p < 0.01
Baby was not okay; was taken to nursery/new born unit/health facility	41	(18.1)	11	(6.0)	p < 0.01
Was born dead/died immediately was born	21	(9.3)	1	(0.5)	p < 0.01
Others	3	(1.3)	0	(0.0)	p < 0.01
Total	227	(100.0)	183	(100.0)	

### Occurrence of obstetric complications and attendant at birth and place of delivery

**Figure 2:**
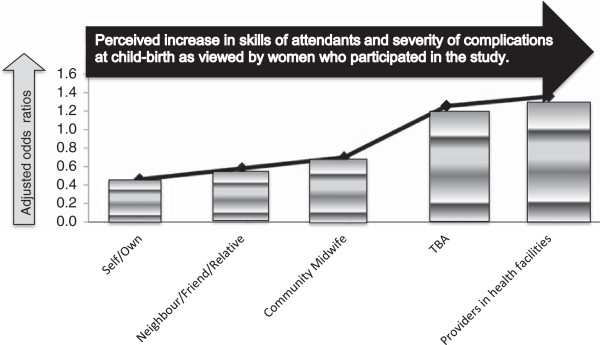
**Risk of occurrence of child-birth related complications by attendants (adjusted odds ratios).**

The risk of occurrence of obstetric complications were higher in facility-based deliveries (adjusted odds ratio ‘AOR’ 1.43 (CI 1.02, 2.01), compared to home deliveries, AOR, 0.70 (CI 0.50, 0.98) p < 0.05. Figure 2 presents a summary of the occurrence and magnitude of obstetric complications and the type of attendant at birth.

The odds of occurrence of obstetric complications appear to be higher among skilled attendants, AOR 1.32 (CI 0.95, 1.84) compared to the non-skilled providers, AOR 0.76 (CI 0.55, 1.05).

## Discussion

The overall objective of the study was to determine the association of provider type and the occurrence and management of major childbirth related complications among postpartum women at the community level. This section highlights important aspects of the results.

### Leading causes of obstetric complications and referral practices

Majority of the respondents said they had experienced severe bleeding or haemorrhage, headache or blurred vision/convulsions or fits (pre-eclampsia/eclampsia), obstructed or prolonged labour, puerperal sepsis and malpresentation or breech presentation. The timing regarding the occurrence of complications shows that the majority of them developed within a very short time (68% within 8 hours and 55% within 0–4 hours). This observation could probably explain why a high proportion of women with complications (45%) sought care within the community. It could be that only life-threatening complications were then referred to health facilities. Some of the women whose complications were managed mentioned that they received drugs and counselling services from community midwives. These are qualified health professionals who reside and operate from their respective communities and majority of them have midwifery and nursing skills. Besides having technical skills, the results also showed that some of the community midwives had the required drugs and other supplies to effectively manage childbirth related complications at the community level.

Investments into such locally available opportunities could help address some of the referral related challenges. Examples of such challenges include long distances travelled to health facilities and other factors that contribute to delays in decision making in seeking care as well as weaknesses in the degree of readiness of health facilities to handle referrals or obstetric complications promptly.

### Main reasons for home deliveries

A high proportion of respondents cited rude staff and undignified care as one of the reasons they preferred home deliveries. Significant differences in the proportion of women who were assisted by non-skilled attendants compared to those who were assisted by skilled attendants preferred TBAs over skilled staff. A higher proportion of women who delivered in health facilities compared to those who delivered at home cited poor quality of care in facilities and high transport costs as some of the reasons many of them prefer home delivery. Whereas, these results were rather unexpected, they probably reflect the personal experiences of many who delivered in health facilities. A recent study conducted in Ghana [16] cited poor attitude of health providers, high delivery and transportation costs and poor quality of care as some of the reasons that made women deliver at home. Unlike the Ghanaian study which sought views only from those who delivered in health facilities, in the Kenyan study, views of study participants on why women in their local community prefer to deliver at home were obtained from both the women who gave birth in health facilities as well as those who delivered at home. Seeking opinions on an important issue from both the users and non-users of a particular service is important in helping to understand different perspectives that exist on the subject and how it shapes the design of interventions. The issue of high delivery costs (which was also cited) is rather baffling given that the study was undertaken long after the Kenya Government had declared free delivery services in the public sector facilities. Delivering at home gives a woman more dignity and is a sign that she is strong. In addition, fear of HIV tests, undergoing unnecessary caesarean-section and mishandling of the placenta were cited as other reasons for delivering at home. Those who talked about the placenta were concerned that the facility staff do discard the placenta in a very careless way instead of burying it the way TBAs do.

### Quality of antenatal care (ANC)

The proportion of women who developed their individualized birth plans was low both in the control arm as well as among the cases. For instance, only 24% of the cases and 25% of the controls sought ANC services at least four times, much lower than the national average of 47% reported in the Kenya Demographic and Health Survey of 2008/2009 cited earlier. A study conducted to measure the impact of birth-preparedness and complication readiness on the use of skilled providers at birth in Burkina Faso established that 46% of pregnant women had a plan for transportation of women in labour to health facilities and 83% had a plan to save money [17]. The results showed that women with these plans were more likely to give birth with the assistance of a skilled provider. These findings highlight how birth-preparedness and complication readiness may be useful in increasing the use of skilled providers at birth.

### Occurrence of obstetric complications

More obstetric complications occurred among the women who were attended to by skilled providers in health facilities during childbirth compared to those who were assisted by unskilled attendants at home (TBAs, neighbours, relatives and friends including self/own deliveries). These results probably imply that either women themselves have a self-selection mechanism or community level attendants tend to refer women they suspect are at a higher risk of developing obstetric complications except for the TBAs who seemed to handle an equally high proportion of complications similar to health facilities. These results need to be interpreted with caution. First, facility based deliveries are extremely low in this region and second, there are negative socio-cultural beliefs and practices which discourage women from delivering in hospitals. Third, there is widespread perception by women and the community members that the quality of care in health facilities is poor and fourth, the study participants complained of high transportation and delivery costs. Thus, given these barriers, it is probable that many of the women attending health facilities do experience life threatening complications with almost no other options to turn to.

The problem of having a high proportion of deliveries being conducted by unskilled personnel is not just confined to Kenya. Despite the high maternal mortality ratio (MMR) of 630/100,000 live births in Nigeria; women predominantly use unskilled attendants at childbirth [18]. A related study examined the nature of the association between maternal mortality and birth with a health professional in observational studies [19]. The results showed little evidence that giving birth with a health professional reduces a woman’s risk of dying, and in some settings it appears to be associated with an increased risk of death. The researchers pointed out that the studies were all conducted in settings where uptake of professional birth attendance is low. Hence, women only seek professional care when they are very sick and perhaps too late for any interventions. Similar observations were made in a recent study that documented the accounts of survivors of life threatening obstetric complications and the barriers to accessing emergency obstetric care in a rural district in Kenya [20]. As expected, most women who had experienced complications were the ones who were delivered by a SBA in the study while those that did not experience any complication probably did not see the need for seeking assistance from a SBA. These findings could explain why the majority of the women without any complication sought the services of non-SBAs.

However, a study conducted in Sri Lanka shows a slightly different scenario [21]. The findings showed a direct correlation between high coverage of SBA and a reduction in maternal mortality ratio. For instance, with SBA coverage of 99 per cent, Sri Lanka’s maternal mortality ratio declined from 340 per 100,000 live births in 1960 to 43 per 100,000 live births in 2005, and 98 per cent of births now take place in hospitals. These results also had positive effects on child survival such that the under-five mortality rate fell from 32 per 1,000 live births in 1990 to 21 per 1,000 live births in 2007 while the neonatal mortality rate fell to 10 per 1,000 live births in 2010 [22].

### Pathways to seeking delivery care and management of obstetric complications

Existing models such as the Three Delay Model [23] and the Health Belief Model [24] cannot adequately explain some of the findings we obtained from women seeking delivery care. The Three Delay Model focuses on factors that affect the interval between the onset of obstetric complication and its outcome with the assumption that if prompt and adequate treatment is provided, the outcome will usually be satisfactory. Although the Three Delay Model seems to emphasise curative interventions, results from the study show that babies who were delivered by women who had obstetric complications were, significantly more likely to be born dead or die immediately they are born (9%) compared to babies who were delivered by women in the control group (0.5%); p < 0.01. These results point towards the need to invest in a public health approach other than to emphasise only on delays and their consequences. We also sought to understand why women in the study community preferred home delivery. Some of the respondents mentioned that giving birth at home is a good sign that the woman is strong while others cited socio-economic status (poverty) among other reasons. It is most likely that the women who said that delivering at home signifies that the woman is strong also thought that pregnancy and labour are not medical issues. These women also thought that pregnancy and labour are natural processes that a woman goes through in the comfort of her home environment. Therefore, applying the Health Belief Model to such groups poses challenges.

Given the weaknesses of the existing models, we developed different scenarios or pathways to help explain the behaviour of women seeking delivery (in a context where skilled attendance during childbirth is low) and the steps they go through once they develop or are at the risk of developing obstetric complications. Probable pathways followed by various women seeking delivery care with or without complications are as follows: First scenario, a woman goes into normal labour and if she does not feel any problem she lets it progress until delivery (self or own delivery). Second scenario: a woman who develops normal labour but she is not confident of how it will progress, invites neighbours/friends or relatives to assist or to keep company. Third scenario: a woman or a third party may choose to go to a community midwife (a skilled provider) for normal delivery or be referred there for further management just in case complications arise or worsen. Fourth scenario: a woman may choose to go to a traditional birth attendant (TBA) for normal delivery or be referred there for further management of her complications. Fifth scenario: a woman may choose to go to a health facility for normal delivery or be referred there for further management of her complications.

A pregnant woman who seeks care interacts with many actors including the care givers. Some of them go into labour and deliver without any problems. Others face many personal, family, community as well as structural level challenges that may act singly or in combination to aggravate their condition.

As a coping mechanism, there are a number of ways a woman uses to deal with barriers and other challenges associated with seeking delivery care:

- She relies on the concept of self-selection, which drives her and her household in choosing where to deliver, who to assist during childbirth and who (where) to go to in case of complications.
- Self-selection is dependent on a number of enabling and constraining factors such as provider attitude, quality of care, cost of seeking care and fear of certain procedures.
- Given the number of barriers women face in seeking facility services, majority of them present or are referred late to these institutions with more serious obstetric complications.

### Limitations of the study

First, the study relied on reports that were provided by women and did not verify the data from facility records. Since the study relied on the mother’s memory, it is probable that some exposure estimates could have been influenced by recall bias. Second, it is probable that cases were more likely to remember details of who their attendants were and how the complications developed than the controls. However, these limitations do not affect the overall results in any significant way since none of the participants knew who was a case and who was a control at the time of the interview. Both the cases and the controls could have been affected equally by possible recall biases. In addition, matching of controls to cases (for age and location) at the design phase of the study also helped address potential confounders in the study.

An assumption was made in the study to the effect that a woman who delivers in a health facility –does so with the help of a skilled birth attendant. We did not observe the attendants conducting deliveries. Rather we relied on the woman’s history and records where appropriate. A review of recent data from the Demographic Health Surveys in Sub-Saharan Africa shows that the proportion of deliveries that occur in health facilities which are not assisted by skilled birth attendants is very low. The implication being that even though we have noted this point as a study limitation, it doesn’t affect in any way the overall findings or conclusions of the study.

## Conclusion

The odds of the occurrence of obstetric complications among the women who were attended to by skilled providers in health facilities during childbirth were greater than the occurrence of similar complications among the unskilled attendants at the community level. It is probable that many of the women who sought care in health facilities presented late or were referred to these institutions with more serious obstetric complications compared to those who sought services elsewhere. Women who suffered from obstetric complications as well as the controls encountered many challenges in seeking care. Whereas some of the challenges are not well understood, others can be addressed through low-cost interventions. Based on findings from the study, we recommend a four-pronged approach in addressing barriers that prevent women from utilising skilled providers for delivery and in managing obstetric complications:

- Prong I- strengthen behavior change communication activities with emphasis on socio-cultural issues and creation of awareness. Activities on creating awareness should emphasise the importance of seeking skilled care promptly. For instance, the odds of the occurrence of obstetric complications among the women who were attended to by TBAs were greater than those who were attended to by community midwives (skilled providers). It is probable that many of the women who sought care from TBAs presented or were referred to them late or simply did not understand the strengths and limitations of this cadre of providers. It is evident that despite their limited kills, TBAs tried to manage a substantial proportion of obstetric complications.
- Prong II – develop and put in place ‘readiness-measures’ at all levels to facilitate provision of first aid and other essential services such as rapid evacuation of women with complications to health facilities using the most cost effective and culturally acceptable means of transport.
- Prong III – improving staff attitude and particularly in dealing with disrespect and abuse as well as the provision of Emergency Obstetric Care services. Availability and accessibility to emergency measures to save the mothers’ lives should go hand in hand with interventions to address perinatal and neonatal morbidity and mortality (results from the study showed that babies who were delivered by women who had obstetric complications were significantly more likely to be born dead or die immediately they are born) compared to babies who were delivered by women in the control group. Provision of complementary services such as food, warm beverages, and bathing water was considered critical by women.
- Prong IV- removing financial barriers and improving coordination of the services at all levels. Local management committees should explore ways of addressing transportation costs and making basic supplies and commodities available in health facilities.

There is also need for:

- Future studies to examine why low proportions of the women had individualised birth plans and to understand further why only a fraction of those women who had the plans delivered at the designated places with their provider of choice.
- Testing out interventions to address the issues around the concept of self-selection in order to assess the effect of eliminating or substantially reducing the barriers on utilisation of health facilities.

## Abbreviations

ANC: Antenatal care
AOR: Adjusted Odds Ratio
BEmOC: Basic Emergency Obstetric Care
C/S: Caesarean section
CEmOC: Comprehensive Emergency Obstetric Care
CI: Confidence Interval
EDD: Expected Date of Delivery
HIV/AIDS: Human Immunodeficiency Virus/Acquired Immune Deficiency Syndrome
ITROMID: Institute of Tropical Medicine and Infectious Diseases
JKUAT: Jomo Kenyatta University of Agriculture and Technology
KEMRI: Kenya Medical Research Institute
MMR: Maternal Mortality Ratio
OR: Odds Ratio
SBA: Skilled Birth Attendant
TB: Tuberculosis
TBAs: Traditional Birth Attendants
UoN: University of Nairobi
WHO: World Health Organization.

## References

[1] Kenya National Bureau of Statistics and ICF Macro (2010). Kenya Demographic and Health Survey 2008–09.

[2] Liambila W, Obare F, Undie C-C, Birungi H, Kuria NS, Wayua RM, Matekwa A (2013). The community midwifery model in Kenya: expanding access to comprehensive reproductive health services. Afr J Midwifery Womens Health.

[3] Alvarez JL, Gil R, Hernández V, Gil A (2009). Factors associated with maternal mortality in Sub-Saharan Africa: an ecological study. BMC Public Health.

[4] World Health Organisation (2010). Background Paper for the Global Strategy for Women’s and Children’s Health.

[5] Canavan A (2009). Review of Global Literature on Maternal Health Interventions and Outcomes Related to Skilled Birth Attendance.

[6] Bisika T (2008). The effectiveness of the TBA Programme in reducing maternal mortality and morbidity in Malawi. East Afr J Public Health.

[7] Republic of Kenya (2013): Office of the Controller of Budget Bungoma County Budget (2013). Implementation Review Report Fourth Quarter FY 2012/2013.

[8] Republic of Kenya -Kenya National Bureau of Statistics, administrative, unit, Kakamega County. Lugari and Likuyani Districts (2009). 2009 Census Results.

[9] Republic of Kenya, Population Council, University of Nairobi (2005). Approaches to Providing Quality Maternal Care.

[10] Ali-Olubandwa AM, Kathuri NJ, Odero-Wanga D, Shivoga WA (2011). Challenges facing small scale maize farmers in Western Province of Kenya in the agricultural reform era 3. Am J Exp Agr.

[11] Khan KS, Wojdyla D, Say L, Gülmezoglu AM, Van Look PF, Khan KS (2006). WHO analysis of causes of maternal death: a systematic review. Lancet.

[12] Hardee K, Gay J, Blanc AK (2012). Maternal morbidity- neglected dimension of safe motherhood in the developing world. Glob Public Health.

[13] World Health Organisation (2003). Managing Complications in Pregnancy and Childbirth: a Guide for Midwives and Doctors.

[14] Suresh KP, Chandrashekara S (2012). Sample size estimation and power analysis for clinical research studies. J Hum Reprod Sci.

[15] Niven DJ, Berthiaume LR, Fick GH, Laupland KB (2012). Matched case–control studies: a review of reported statistical methodology. Clin Epidemiol.

[16] Esena RK, Sappor MM (2013). Factors Associated with the utilization of skilled delivery services in the Ga East Municipality of Ghana Part 2: barriers to skilled delivery. Int J Sci Tech Res.

[17] Moran AC, Moran AC, Sangli G, Dineen R, Rawlins B, Yaméogo M, Baya B (2006). Birth-preparedness for maternal health: findings from Koupéla District, Burkina Faso. J Health Popul Nutr.

[18] Fapohunda BM, Orobaton NG (2013). When women deliver with no one present in Nigeria: who, what, where and so what?. PLoS One.

[19] Scott S, Ronsmans C (2009). The relationship between birth with a health professional and maternal mortality in observational studies: a review of the literature. Trop Med Int Health.

[20] Echoka E, Makokha A, Dubourg D, Kombe Y, Nyandieka L, Byskov J (2014). Barriers to emergency obstetric care services: accounts of survivors of life threatening obstetric complications in Malindi District, Kenya. Pan Afr Med J.

[21] UNICEF: *Prioritizing Maternal Health in Sri Lanka*. http://www.unicef.org/devpro/46000_48498.html

[22] UNICEF (2012). Maternal, Newborn & Child Survival Prioritizing maternal health in Sri Lanka.

[23] Thaddeus S, Maine D (1994). Too far to walk: maternal mortality in context. Soc Sci Med.

[24] Karen G, Rimer BK, Viswanath K (2008). Health behaviour and health education: theory, research, and practice.

[CR25] The pre-publication history for this paper can be accessed here:http://www.biomedcentral.com/1471-2393/14/311/prepub

